# Supplementary material for: Structural basis for the non-self RNA-activated protease activity of the type III-E CRISPR nuclease-protease Craspase
Source: Nat Commun. 2022 Dec 7;13:7549. doi: 10.1038/s41467-022-35275-5 (PMC9729208; doi:10.1038/s41467-022-35275-5)
Supplement: Supplementary file 1 — Supplementary Information [file 41467_2022_35275_MOESM1_ESM.pdf]

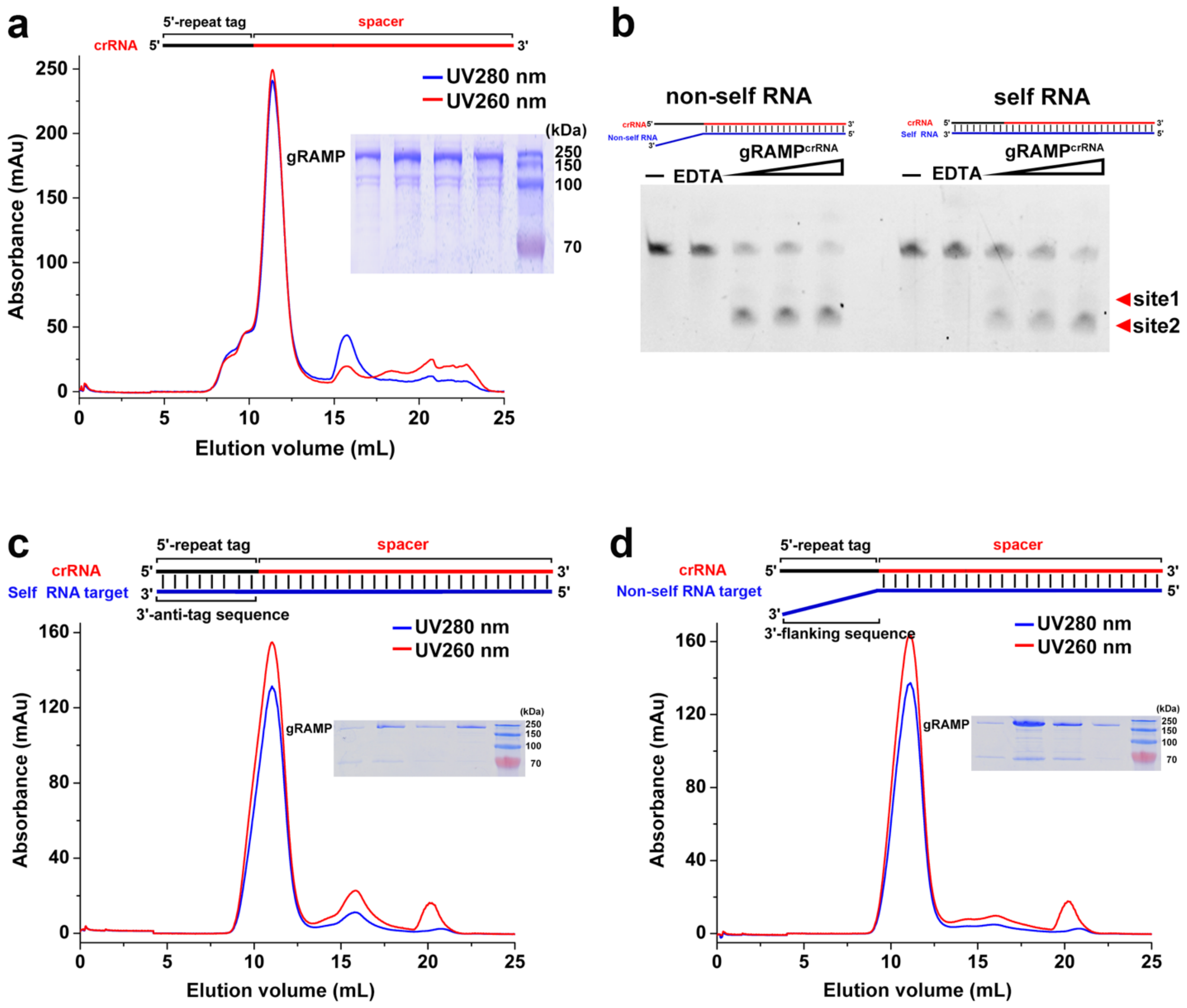

Supplementary Fig. 1 Biochemical reconstruction of *Candidatus* "Scalindua brodae" gRAMP^{crRNA} binary, gRAMP^{crRNA}-target RNA^{self} and gRAMP^{crRNA}-target RNA$^{non\text{-}self}$ ternary complexes. a Size-exclusion chromatography, and SDS-PAGE profile for purification of gRAMP^{crRNA} binary complex. **b** Cleavage of target RNA by gRAMP^{crRNA} complex. Increasing concentrations (100, 200, 400 nM) of gRAMP^{crRNA} complex were incubated with 50 nM FAM-labeled non-self or self RNA targets at 37°C for 60 mins, respectively. *In vitro* RNA cleavage experiments were repeated at least three times with similar results. **c** and **d** Size-exclusion chromatography, and SDS-PAGE profile for *in vitro* assembly of gRAMP^{crRNA} complex with either self RNA (**c**) or non-self (**d**) RNA targets. Red and blue curves correspond to 260 and 280 nm UV absorptions, respectively.

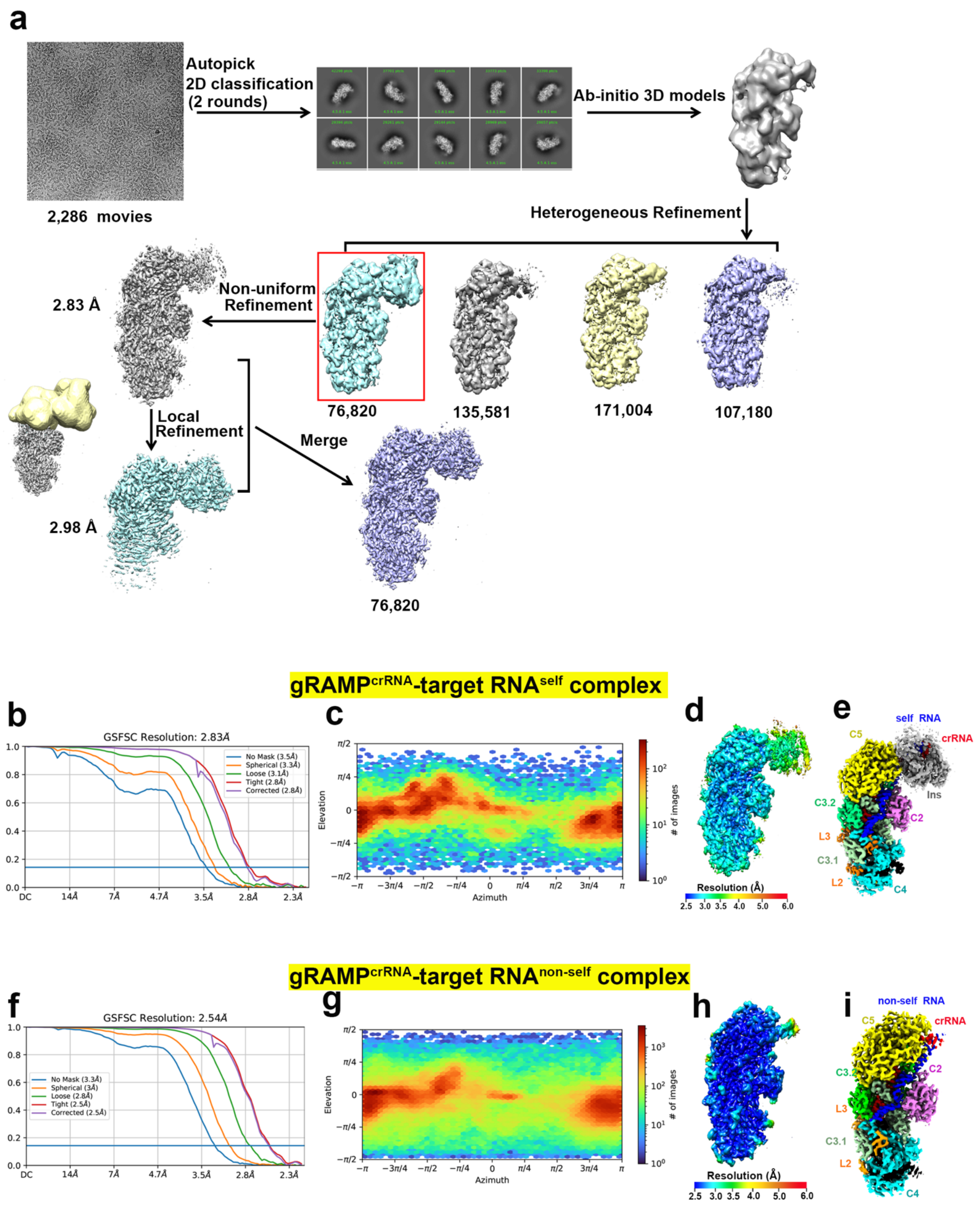

Supplementary Fig. 2 Cryo-EM reconstruction of gRAMP^{crRNA}-target RNA^{self} and gRAMP^{crRNA}-target RNA$^{non\text{-}self}$ ternary complexes. a Flow chart of image processing for gRAMP^{crRNA}-target RNA^{self} ternary complex. **b** and **f** Fourier Shell Correlation (FSC) curve of gRAMP^{crRNA}-target RNA^{self} (**b**) and gRAMP^{crRNA}-target RNA$^{non\text{-}self}$ (**f**) ternary complex

reconstruction. **c** and **g** Direction distribution plot of $gRAMP^{crRNA}$-target RNA^{self} (**c**) and $gRAMP^{crRNA}$-target $RNA^{non\text{-}self}$ (**g**) ternary complex reconstruction. **d** and **h** Final 3D reconstructed map of $gRAMP^{crRNA}$-target RNA^{self} (**d**) and $gRAMP^{crRNA}$-target $RNA^{non\text{-}self}$ (**h**) ternary complex colored according to local resolution. **e** and **i** cryo-EM reconstruction of $gRAMP^{crRNA}$-target RNA^{self} (**e**) and $gRAMP^{crRNA}$-target $RNA^{non\text{-}self}$ (**i**) complexes.

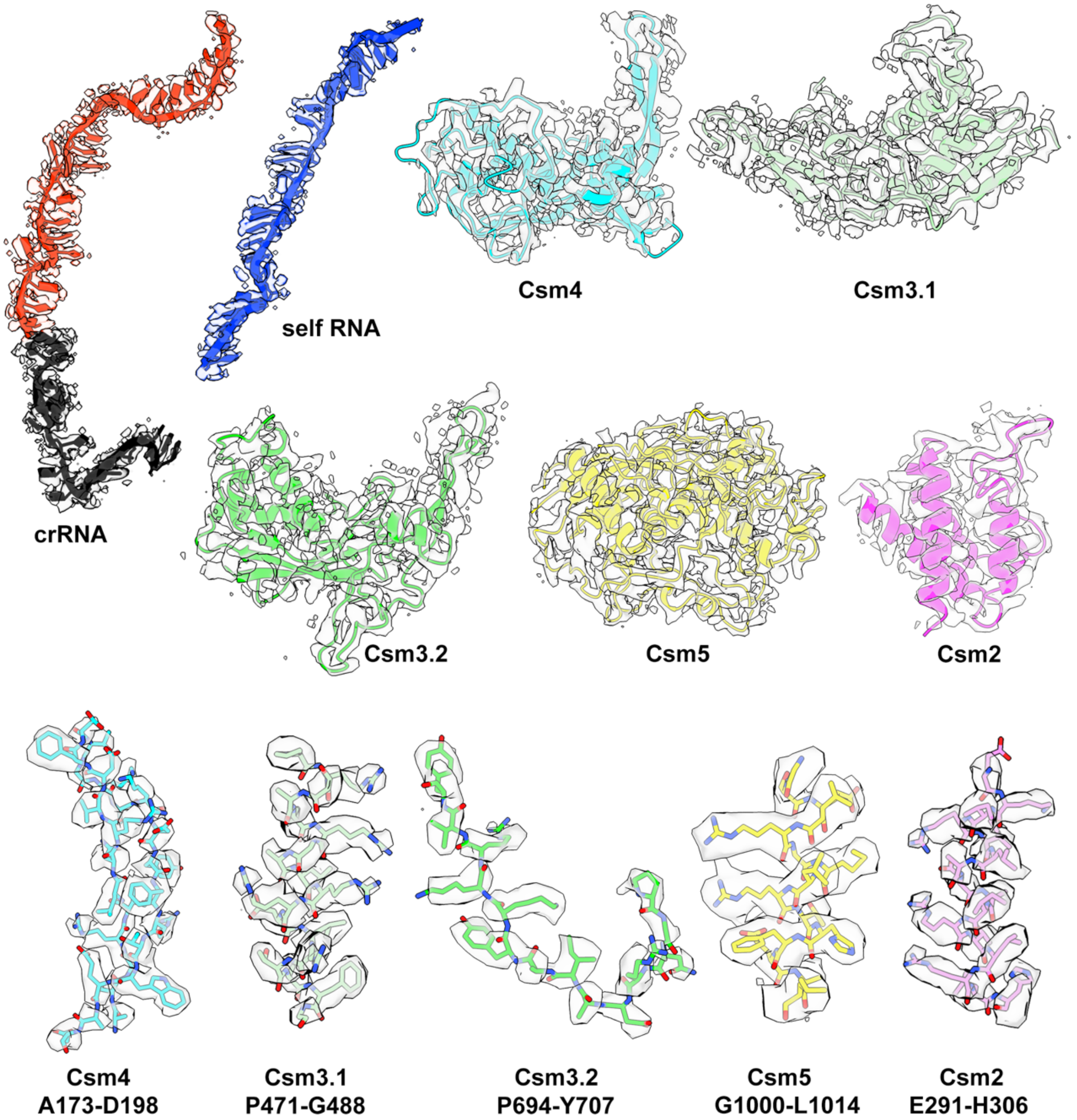

Supplementary Fig. 3 Cryo-EM density of the gRAMP^{crRNA}-target RNA^{self} structure. Densities for indicated regions are shown in the context of the atomic model.

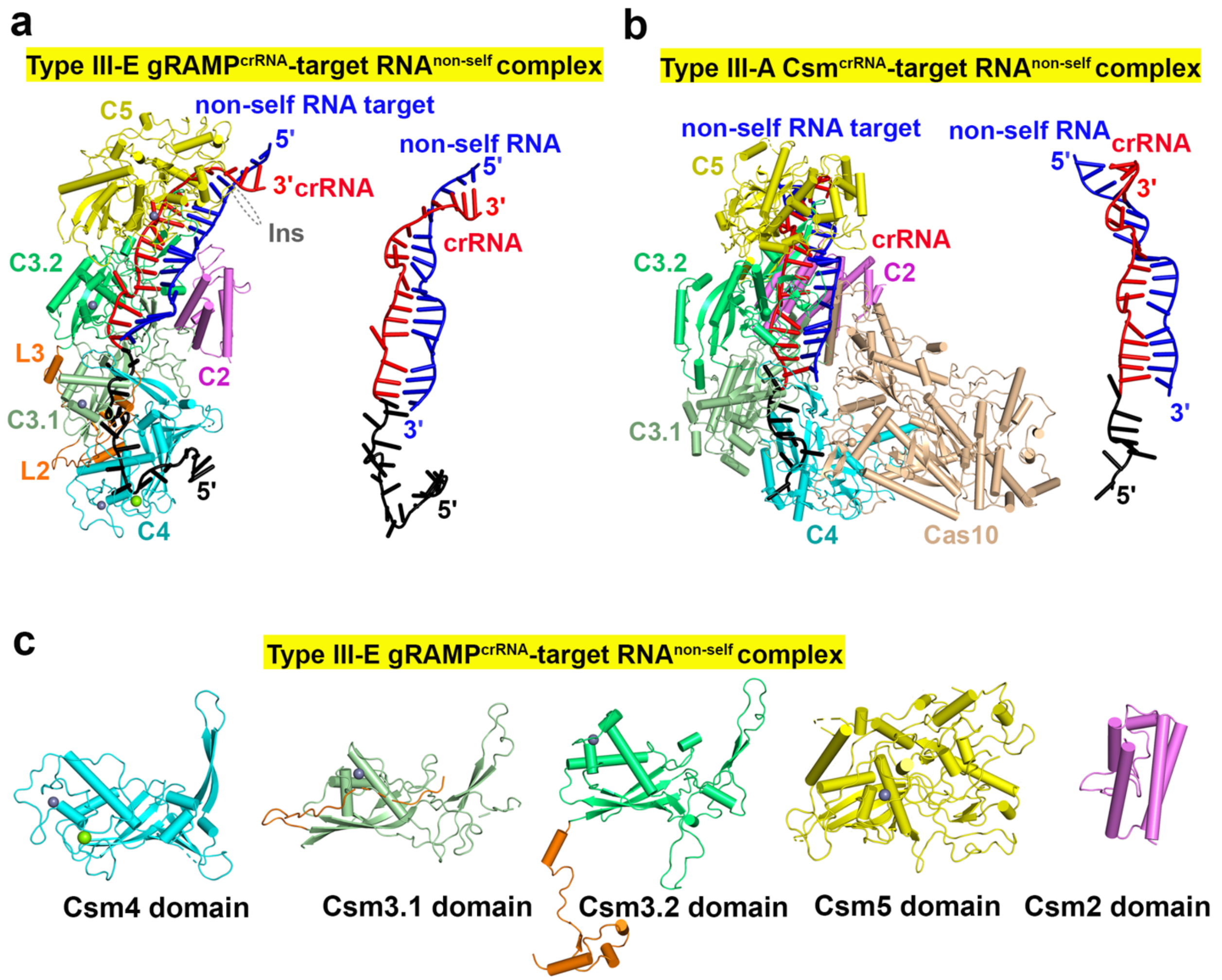

Supplementary Fig. 4 Structural comparison between type III-E gRAMP^{crRNA}-target RNA$^{non\text{-}self}$ and type III-A Csm^{crRNA}-target RNA$^{non\text{-}self}$ complexes. a and **b**, Cryo-EM structure of type III-E gRAMP^{crRNA}-target RNA$^{non\text{-}self}$ complex (**a**), Csm^{crRNA}-target RNA$^{non\text{-}self}$ complex (**b**, PDB 6MUR) and its crRNA-target RNA$^{non\text{-}self}$ duplex. **c** and **d**, Individual subunits of the type III-E gRAMP (**c**) and type III-A Csm (**d**) complexes.

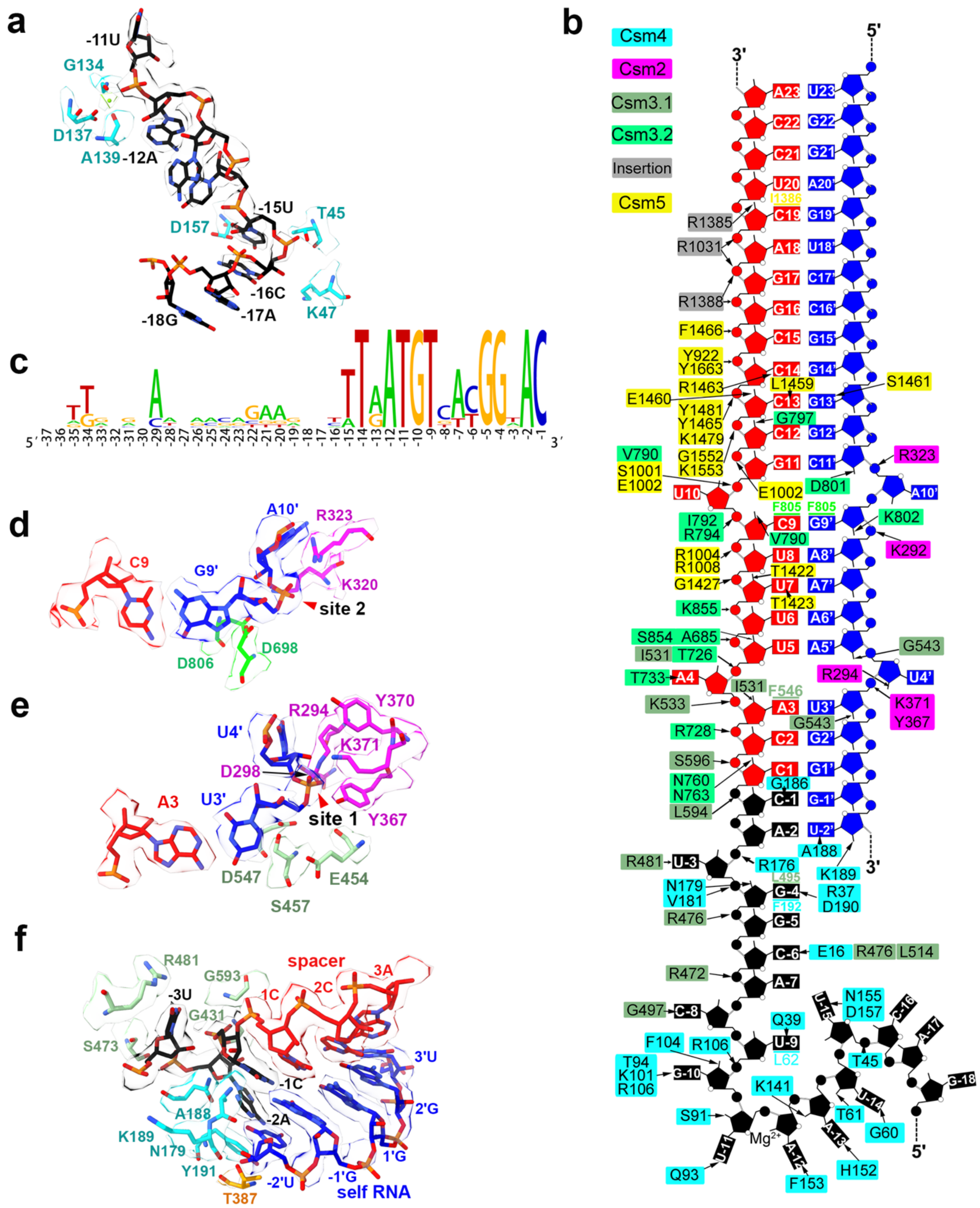

Supplementary Fig. 5 Recognition of crRNA-target RNA duplex by gRAMP. a, d, e and f Cryo-EM density for indicated regions in the gRAMP^{crRNA}-target RNA^{self} structure in Fig. 2b. **b** Detailed interactions between crRNA-target RNA duplex and gRAMP in gRAMP^{crRNA}-target RNA$^{non-self}$ ternary complex. **c** Seqlogos of representative type III-E repeats constructed by WebLogo (http://weblogo.berkeley.edu/logo.cgi). Repeats are found by CRISPRminer (http://www.microbiome-bigdata.com/CRISPRminer) from MGTA01000040.1;

PDWI01005922.1; SESD01000293.1; OBJA01001127.1; NZ_BEXT01000001.1; RHLA01000020.1; JRYO01000185.1.

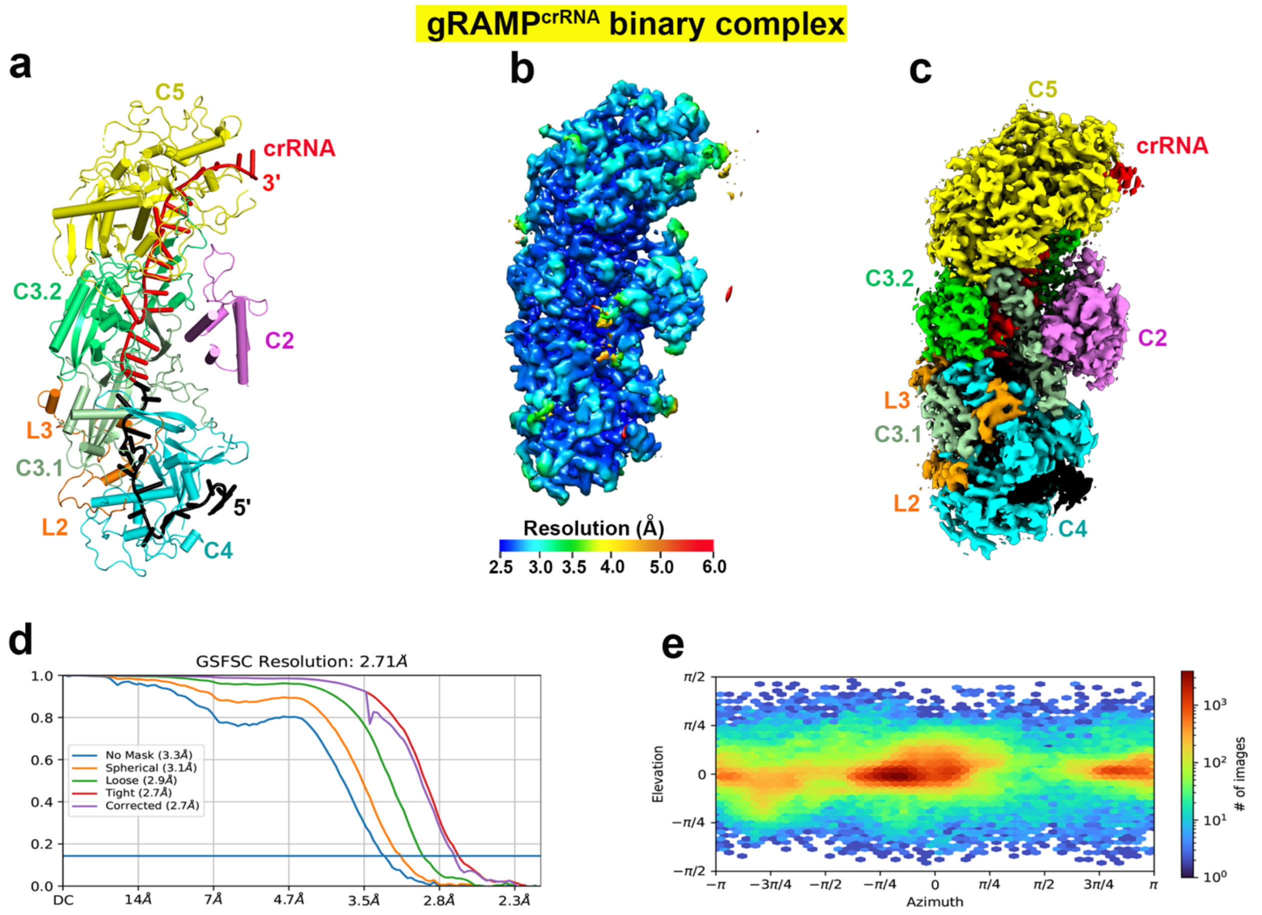

Supplementary Fig. 6 Cryo-EM Reconstruction of gRAMP^{crRNA} binary complex. a Cryo-EM structure of gRAMP^{crRNA} binary complex. **b** Final 3D reconstructed map of gRAMP^{crRNA}-binary complex colored according to local resolution. **c** cryo-EM reconstruction of gRAMP^{crRNA}-binary complex. **d** FSC curve of gRAMP^{crRNA} binary complex reconstruction. **e** Direction distribution plot of gRAMP^{crRNA} binary complex reconstruction.

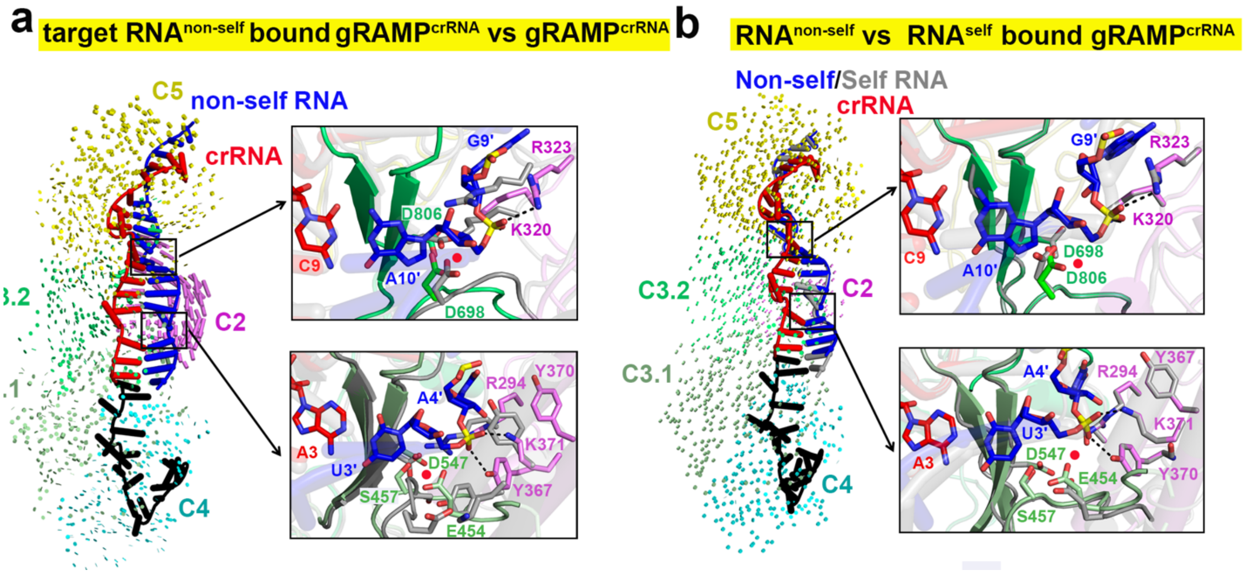

Supplementary Fig. 7 Structural comparison of RNase catalytic pockets in gRAMP complexes. **a**, Structure comparison of the overall structure and residues in the RNase catalytic pockets in gRAMP^{crRNA} binary (in grey) and gRAMP^{crRNA}-target RNA$^{non\text{-}self}$ ternary complex (in color). **b**, Structure comparison of the overall structure and residues in the RNase catalytic pockets in gRAMP^{crRNA}-target RNA^{self} (in grey) and gRAMP^{crRNA}-target RNA$^{non\text{-}self}$ ternary complexes (in color).

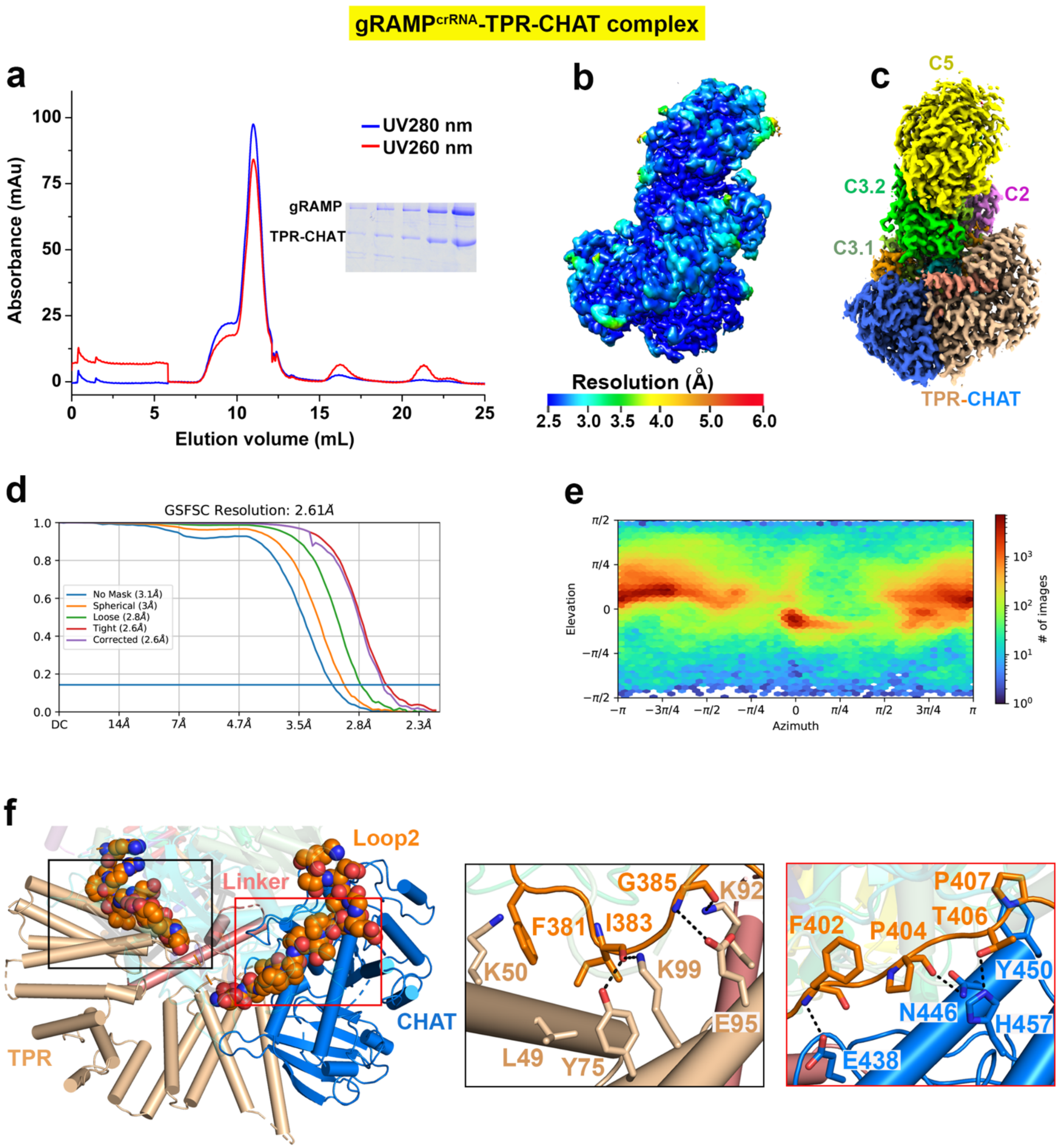

Supplementary Fig. 8 Reconstruction of gRAMP^{crRNA}-TPR-CHAT complex. a Size-exclusion chromatography, and SDS-PAGE profile for purification of gRAMP^{crRNA}-TPR-CHAT complex. Red and blue curves correspond to 260 and 280 nm UV absorptions, respectively. **b** Final 3D reconstructed map of gRAMP^{crRNA}-TPR-CHAT complex colored according to local resolution. **c** cryo-EM reconstruction of gRAMP^{crRNA}-TPR-CHAT binary complex. **d** FSC curve of gRAMP^{crRNA}-TPR-CHAT complex reconstruction. **e** Direction distribution plot of gRAMP^{crRNA}-TPR-CHAT complex reconstruction. **f** The detailed interactions between TPR-CHAT and Loop 2 in gRAMP in gRAMP^{crRNA}-TPR-CHAT complex.

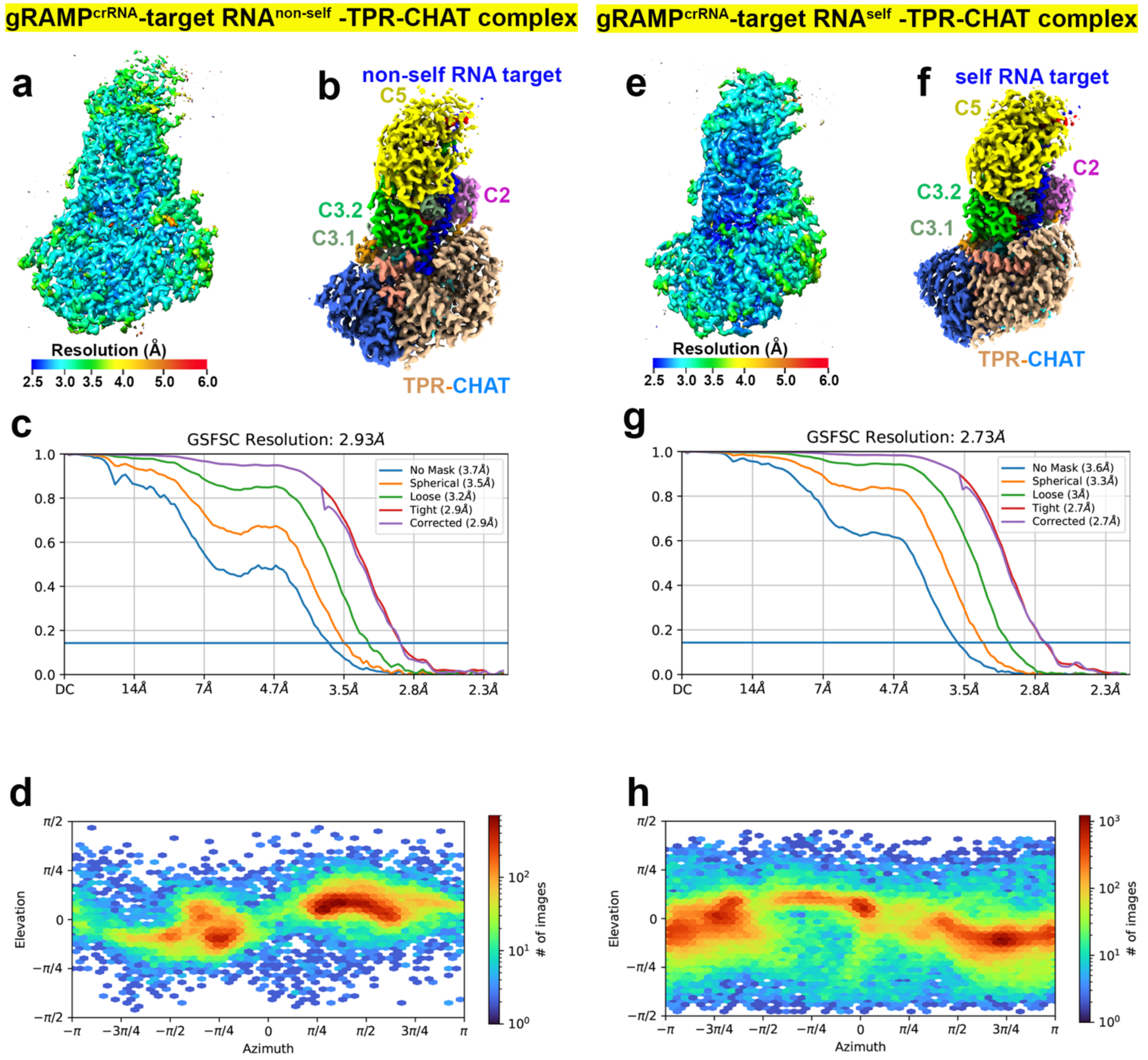

Supplementary Fig. 9 Cryo-EM Reconstruction of target RNA bound gRAMP^{crRNA}-TPR-CHAT complex. a and **e** Final 3D reconstructed map of gRAMP^{crRNA}-TPR-CHAT-target RNA$^{\text{non-self}}$ complex (**a**) and gRAMP^{crRNA}-TPR-CHAT-target RNA^{self} complex (**e**) colored according to local resolution. **b** and **f** cryo-EM reconstruction of gRAMP^{crRNA}-TPR-CHAT-target RNA$^{\text{non-self}}$ (**b**) and gRAMP^{crRNA}-TPR-CHAT-target RNA^{self} complex (**f**). **c** and **g** FSC curve of gRAMP^{crRNA}-TPR-CHAT-target RNA$^{\text{non-self}}$ complex (**c**) and gRAMP^{crRNA}-TPR-CHAT-target RNA^{self} complex (**g**) reconstruction. **d** and **h** Direction distribution plot of gRAMP^{crRNA}-TPR-CHAT-target RNA$^{\text{non-self}}$ (**d**) and gRAMP^{crRNA}-TPR-CHAT-target RNA^{self} (**h**) complex reconstruction.

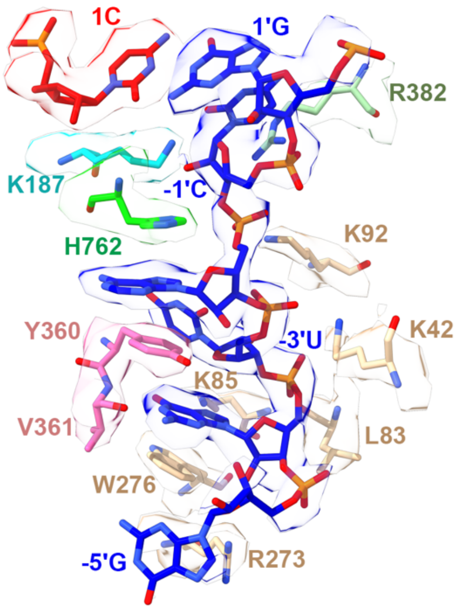

Supplementary Fig. 10 Cryo-EM density for indicated regions in the gRAMP^{crRNA}-target RNA^{self} structure in Fig. 4c.

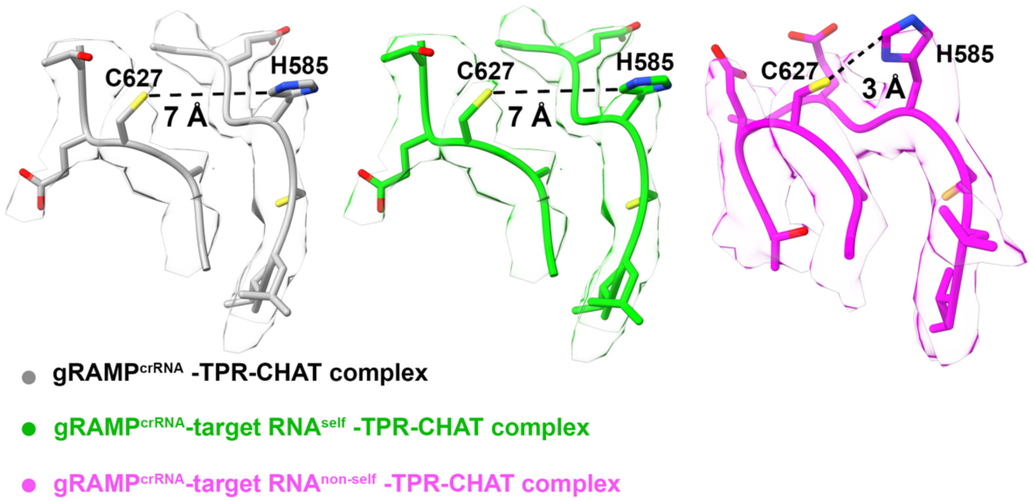

Supplementary Fig. 11 Cryo-EM density of catalytic residues H585 and C627 in different gRAMP-TPR-CHAT complexes.

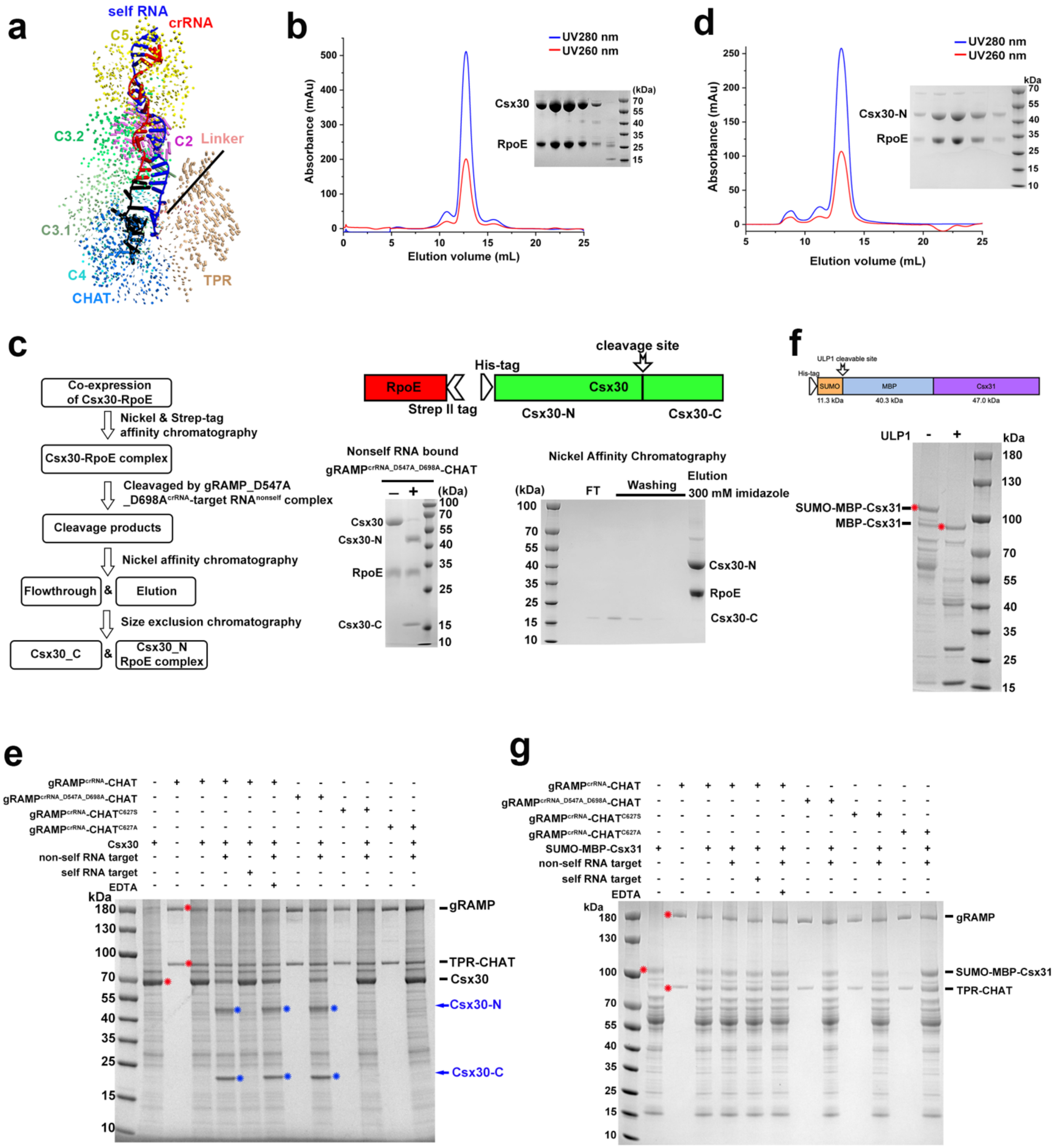

Supplementary Fig. 12 The protease activity of gRAMP^{crRNA}-TPR-CHAT complex. a Structure comparison between gRAMP^{crRNA}-TPR-CHAT before and after self RNA target binding. Vector length indicates the domain movement scale. **b** and **d** The gel filtration file of Csx30-RpoE complex (**b**) and Csx30-N-RpoE complex (**d**). **c** The flow chart and nickel affinity chromatography assay for purification of Csx30-N-RpoE complex after Csx30 cleavage. FT indicates Flow through. **e** and **g** Cleavage of Csx30 (**e**) or SUMO-MPB-Csx31 protein (**g**) by the gRMAP^{crRNA}-CHAT complex and its mutants. 1 μM gRAMP^{crRNA}-TPR-CHAT or its mutants was incubated with 5 μM

Csx30 or SUMO-MPB-Csx31 at 37 °C for 60 mins. The red asterisk indicates the position of input proteins. **f** Cleavage Csx31fused with ULP1 Maltose binding protein (MBP) and SUMO tags demonstrated the expression and position of SUMO-MPB-Csx31 protein. We could only get the soluble Csx31 fused with SUMO and MPB tags. *In vitro* cleavage experiments were repeated at least three times with similar results.

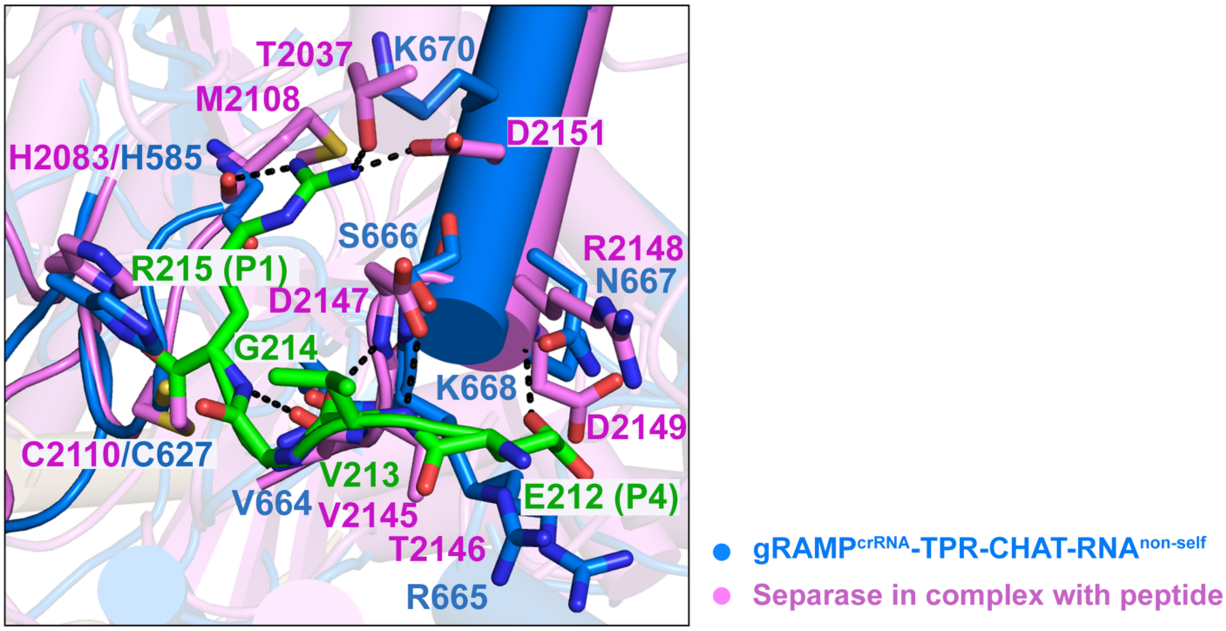

Supplementary Fig. 13 Superposition of residues in the catalytic pockets between TPR-CHAT (in marine) and *Chaetomium thermophilum* separase (in violet, PDB 5FC3).

Supplementary Table 1. Cryo-EM data collection, refinement and validation statistics

	gRAMP^{crRNA}	gRAMP^{crRNA}-target RNA$^{non-self}$	gRAMP^{crRNA}-target RNA^{self}	gRAMP^{crRNA}-TPR-CHAT	gRAMP^{crRNA}-TPR-CHAT-target RNA$^{non-self}$	gRAMP^{crRNA}-TPR-CHAT-target RNA^{self}
**Data collection and processing**						
Microscope	FEI Titan Krios	FEI Titan Krios	FEI Titan Krios	FEI Titan Krios	FEI Titan Krios	FEI Titan Krios
Camera	Gatan K3	Gatan K3	Gatan K3	Gatan K3	Gatan K3	Gatan K3
Magnification	105,000	105,000	105,000	105,000	105,000	105,000
Voltage (kV)	300	300	300	300	300	300
Total electron exposure (e^{-}/Å^2)	50	50	50	50	50	50
Number of frames	32	32	32	32	32	32
Defocus range (μm)	-1.5 ~-2.5	-1.5 ~-2.5	-1.5 ~-2.5	-1.5 ~-2.5	-1.5 ~-2.5	-1.5 ~-2.5
Pixel size (Å/pixel)	1.1	1.1	1.1	1.1	1.1	1.1
Software	RELION 3.1, cryoSPARC v2	RELION 3.1, cryoSPARC v2	RELION 3.1, cryoSPARC v2	RELION 3.1, cryoSPARC v2	RELION 3.1, cryoSPARC v2	RELION 3.1, cryoSPARC v2
Symmetry imposed	C1	C1	C1	C1	C1	C1
Initial particle images (no.)	2,895,967	3,310,582	1,610,246	3,120,917	1,792,636	2,002,116
Final particle images (no.)	227,997	444,936	76,820	611,350	53,439	123,862
Overall map resolution (Å)	2.71	2.54	2.83	2.61	2.93	2.73
FSC threshold	0.143	0.143	0.143	0.143	0.143	0.143
Local map resolution range (Å)	2.5~3.5	2.5~3.5	2.5~4.0	2.5~3.5	2.5~4.0	2.5~3.5
**Refinement**						
**Software**	Phenix 1.13 realspace-refine	Phenix 1.13 realspace-refine	Phenix 1.13 realspace-refine	Phenix 1.13 realspace-refine	Phenix 1.13 realspace-refine	Phenix 1.13 realspace-refine
**Symmetry imposed**	C1	C1	C1	C1	C1	C1
Initial model used (PDB code)	*ab-initio*	*ab-initio*	*ab-initio*	*ab-initio*	*ab-initio*	*ab-initio*
Model resolution (Å)	2.91	2.72	3.05	2.73	3.17	2.96
FSC threshold	0.5	0.5	0.5	0.5	0.5	0.5
Map sharpening *B* factor (Å^2)	-110.6	-106.6	-98.6	-119.3	-85.2	-96.5
Map Correlation Coefficient	0.75	0.80	0.82	0.82	0.80	0.75
Model composition						
Non-hydrogen atoms	10,572	11,044	12,278	16,081	16,292	16,502
Protein residues	1,218	1,223	1,341	1,894	1,860	1,884
Nucleotide base	35	55	66	35	59	59
B factor (Å^2)						
Protein	54.58	43.72	32.54	38.54	52.78	94.20
Nucleotide	51.10	47.35	45.25	40.12	63.31	93.04
R.m.s. deviations						
Bond lengths (Å)	0.005	0.005	0.003	0.006	0.003	0.004
Bond angles (°)	0.883	0.784	0.576	0.644	0.571	0.572
Validation						
MolProbity score	1.89	1.72	2.19	1.91	2.50	2.21
Clash score	8.42	6.73	15.97	14.68	17.67	15.48
Poor rotamers (%)	3.77	2.91	4.34	2.04	5.46	3.93

Ramachandran plot						
Favored (%)	98.24	98.75	98.25	98.06	96.87	97.73
Allowed (%)	1.76	1.25	1.75	1.94	3.13	2.27
Disallowed (%)	0	0	0	0	0	0
PDB	7Y80	7Y81	7Y82	7Y84	7Y83	7Y85
EMDB	33676	33677	33678	33680	33679	33681
